# Ergonomics and Exercise Training as a Basic Intervention for Work-Related Musculoskeletal Disorders in Construction Workers: A Randomized Controlled Trial

**DOI:** 10.3390/healthcare14142235

**Published:** 2026-07-22

**Authors:** Ömer Şevgin, Betül Berat Yıldız, Ertuğrul Safran

**Affiliations:** 1 Department of Physiotherapy and Rehabilitation, Faculty of Health Sciences, Üsküdar University, İstanbul 34768, Türkiye; betul.yildiz0104@gmail.com; 2 Department of Physiotherapy and Rehabilitation, Faculty of Health Sciences, Bezmialem Vakıf University, İstanbul 34093, Türkiye; esafran@bezmialem.edu.tr

**Keywords:** construction workers, exercise, musculoskeletal disorders, occupational health, public health

## Abstract

**Background:** Work-related musculoskeletal disorders (WRMSDs) are highly prevalent among construction workers. Ergonomics training and exercise are both used for their management, but the incremental benefit of adding exercise to ergonomics training in this population is unclear. Our aim was to evaluate whether adding a supervised exercise programme to ergonomics training provides additional benefit over ergonomics training alone in construction workers with WRMSDs. **Methods:** In this single-centre, two-arm randomized controlled trial, 52 male construction workers with musculoskeletal complaints were allocated to an ergonomics training plus exercise group (n = 26) or an ergonomics training alone group (active comparator, n = 26) for 12 weeks. The primary outcome was pain intensity (Visual Analogue Scale—VAS); secondary outcomes were musculoskeletal symptom burden (Extended Nordic Musculoskeletal Questionnaire, NMQ-E), fatigue (Fatigue Severity Scale—FSS), sleep quality (Pittsburgh Sleep Quality Index—PSQI), and occupational burnout (Maslach Burnout Inventory—MBI). The pre-specified primary contrast was the baseline-adjusted between-group difference at 12 weeks (ANCOVA), with a responder analysis as a sensitivity check. **Results:** Both groups improved significantly from baseline in terms of pain, fatigue, sleep quality, and musculoskeletal symptom burden (all *p* < 0.001) and in personal accomplishment (*p* < 0.001); emotional exhaustion and depersonalization were unchanged in both arms. Baseline-adjusted between-group comparisons showed no statistically significant incremental effect of added exercise on pain (adjusted difference −0.34, 95% CI −0.99 to 0.32), fatigue, sleep, symptom burden, or any burnout dimension. The exercise arm showed numerically greater improvements in pain, fatigue, and symptom burden and a higher proportion achieving ≥30% pain relief (96% vs. 77%; odds ratio 7.5), but these differences did not reach statistical significance (*p* = 0.099). **Conclusions:** Over a period of 12 weeks, ergonomics training with or without added exercise was associated with significant improvements in musculoskeletal and related outcomes; however, adding exercise to ergonomics training produced no statistically significant incremental benefit. A non-significant trend toward greater pain response with added exercise warrants confirmation in larger, longer trials.

## 1. Introduction

Musculoskeletal disorders (MSDs) are among the most prevalent non-communicable conditions worldwide and constitute one of the leading contributors to years lived with disability, affecting more than 1.6 billion people globally [1]. Low back pain is the most disabling of these conditions: an estimated 619 million people were living with low back pain in 2020—a figure projected to rise to 843 million by 2050—and it remains the single leading cause of disability across most world regions [2]. When MSDs are caused or aggravated by occupational exposures, they are referred to as work-related musculoskeletal disorders (WRMSDs). WRMSDs are commonly associated with repetitive movements, sustained non-neutral postures, forceful exertion, and manual material handling [3,4].

The construction sector is consistently identified as one of the highest-risk occupational environments for WRMSDs. A recent meta-analysis estimated an overall WRMSD prevalence of 59%, with the lower back and shoulders being the most frequently affected regions; findings regarding demographic risk factors were inconsistent and highly heterogeneous [5]. Comparative occupational data have long shown that construction workers carry a disproportionate burden of musculoskeletal morbidity and work disability relative to other sectors [6,7]. This excess risk reflects the biomechanical demands intrinsic to construction work, including overhead assembly requiring prolonged shoulder elevation, hand–arm vibration from power tools, repetitive precision tasks, squatting and kneeling, and the frequent manual handling of heavy and unstable loads [8].

These exposures repeatedly load the musculoskeletal system beyond its physiological tolerance, producing deviations from neutral posture that progress to physical strain and, ultimately, functional limitation [3]. Beyond their direct effect on workers’ health, WRMSDs impose a substantial economic and occupational burden through reduced productivity, sickness absence, premature work disability, and elevated healthcare expenditure [6,9]. For physically demanding sectors such as construction, this establishes a clear imperative for preventive and rehabilitative strategies that are both clinically effective and feasible to deliver in the field.

Two complementary approaches dominate the management of work-related musculoskeletal complaints: therapeutic exercise and ergonomic intervention. Exercise has moderate supporting evidence for chronic spinal pain and improves pain and function in mechanical neck disorders, although effect sizes are variable [10,11]. Ergonomic approaches may range from education on body mechanics and safe working practices to comprehensive workplace interventions involving task redesign, equipment adjustment, participatory assessment, and physical modification of occupational exposure [9]. These approaches should not be considered equivalent, because education may improve workers’ knowledge and behaviour, whereas workplace modification directly targets biomechanical risk factors. The present study evaluated an education-based ergonomics programme and did not include physical modification of the workplace, tools, or job tasks. There is growing interest in combining these approaches, with the rationale that exercise targets individual physical capacity while ergonomic training targets the work environment and behaviour. However, the evidence base remains heterogeneous, and few trials have rigorously isolated the added value of one component over the other in high-risk manual sectors.

Methodologically robust randomized controlled trials targeting construction workers specifically—rather than office or sedentary populations [12]—remain scarce, and the incremental benefit of adding a structured exercise programme to ergonomics training has not been clearly established in this group. Construction workers represent a particularly important population for workplace prevention because their occupational exposure combines heavy manual handling, repetitive movements, non-neutral postures, vibration, and prolonged physical loading. At the same time, long working hours and physically demanding tasks may limit recovery and reduce the feasibility of additional workplace exercise. Consequently, it is clinically and operationally important to determine whether adding a structured exercise programme to basic ergonomics training produces sufficient incremental benefit to justify the additional time, supervision, and adherence requirements. However, this specific comparative question has rarely been examined in randomized trials involving construction workers. The present study was therefore designed to determine whether adding a supervised exercise programme to ergonomics education provides additional benefit over ergonomics education alone in construction workers with persistent musculoskeletal complaints. We hypothesized that the ergonomics-plus-exercise group would show greater improvement than the ergonomics-only group; pain intensity was the primary outcome and musculoskeletal symptom burden, fatigue, sleep quality, and burnout were secondary outcomes.

## 2. Materials and Methods

### 2.1. Study Design and Ethics

This was a single-centre, two-arm, parallel-group randomized controlled trial conducted between September 2025 and February 2026 and reported in accordance with the CONSORT 2010 statement [13]. The trial was designed as an active-comparator study to evaluate the incremental effect of adding supervised exercise to ergonomics training rather than the absolute effect of either intervention compared with no treatment. The study was approved by the Non-Interventional Research Ethics Committee of Üsküdar University (meeting no. 09, 27 August 2025; decision no. 61351342/020-70) and was conducted in accordance with the Declaration of Helsinki [14]. Written informed consent was obtained from all participants. The protocol is registered with http://clinicaltrials.gov/ (28 August 2025, Clinical Trial, NCT07157800).

### 2.2. Participants and Sample Size

**Figure 1:**
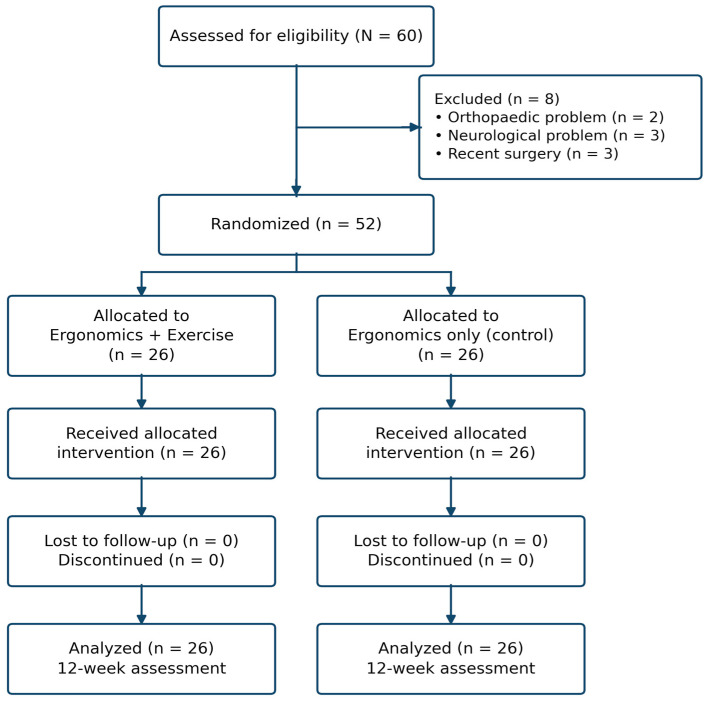
CONSORT participant flow diagram.

Participants were male construction workers employed at a single construction company who reported musculoskeletal pain and/or functional loss. Sample size was estimated using G*Power 3.1 (Heinrich Heine University Düsseldorf, Düsseldorf, Germany) [15]. The original calculation was based on a two-group, two-timepoint repeated-measures model, assuming a large effect size (f = 0.41), a two-sided type-I error rate of 0.05, and 95% statistical power, which indicated a minimum required sample of 37 participants [16,17]. However, the calculation was not specifically based on an expected baseline-adjusted between-group difference in the primary VAS outcome. Therefore, the study was designed to detect a relatively large group-by-time effect and may have had insufficient statistical power to detect small or moderate incremental effects of adding exercise. Sixty workers were initially assessed; eight were excluded (two for orthopedic problems, three for neurological problems, and three for surgery within the preceding year), leaving 52 participants (Figure 1).

### 2.3. Randomization and Blinding

The 52 eligible participants were allocated 1:1 to the two groups using simple randomization, yielding 26 participants per arm. Participants were randomly assigned to the intervention or control group using the sequentially numbered, opaque, sealed envelope (SNOSE) method to ensure allocation concealment. The random allocation sequence was generated before participant enrollment by an independent researcher who was not involved in participant recruitment, intervention delivery, or outcome assessment. Sequentially numbered, opaque, sealed envelopes containing the group assignments were prepared according to the randomization sequence. After confirming participant eligibility and obtaining written informed consent, the next envelope in the sequence was opened to reveal the group assignment. Further details regarding sequence generation and allocation concealment were not prospectively documented; therefore, no claim of concealed allocation is made, and this issue is acknowledged as a potential source of selection bias. Owing to the nature of the interventions, participants and the treating physiotherapist could not be blinded. The same physiotherapist delivered the interventions and administered the outcome questionnaires; therefore, outcome collection was not assessor-blinded. The dataset was coded and analyzed without group-identifying labels to reduce data-handling and analytic bias, providing analyst masking only. However, this procedure could not eliminate expectation, performance, reporting, or detection bias associated with the unblinded delivery and collection of participant-reported outcomes.

### 2.4. Inclusion and Exclusion Criteria

Participants were eligible if they were male construction workers aged 25–55 years; had musculoskeletal pain with a VAS score of at least 3; reported associated functional limitation that did not completely restrict activities of daily living; had experienced low-back, knee, neck, wrist, or shoulder pain for at least three months; worked at least 45 h per week; and provided written informed consent. Participants were excluded if they had an orthopedic or neurological condition that contraindicated exercise, had undergone surgery within the preceding year, or had a psychiatric condition considered likely to substantially affect pain perception or participation. Exclusion criteria were any orthopedic or neurological condition precluding exercise, and any psychiatric condition (e.g., depression, anxiety) liable to affect pain perception. Participants could discontinue the study at their own request or if they developed a new medical condition that prevented safe participation in the intervention.

### 2.5. Interventions

All assessments and interventions were conducted by an experienced physiother-apist. Both groups received an individual ergonomics education programme, while the experimental group also participated in a supervised exercise programme. Ergonomics education was delivered individually on three occasions—at baseline, at the end of week 4, and at the end of week 8—with each session lasting approximately two hours. The educational content included the definition and principles of ergonomics, occupational risk factors, recommended ergonomic modifications, prevention of musculoskeletal complaints, correct body mechanics during work, appropriate rest intervals, safe and appropriate use of work equipment, and occupational safety and efficiency. The ergonomics component was education-based and did not include physical modification of the workplace, redesign of work tasks, or adjustment of tools or equipment. The ergonomics training program was delivered using standardized educational materials, including visual slide presentations and instructional videos. These sessions focused on specific behavioural targets, such as maintaining optimal seated posture, organizing the workstation, and applying joint-protection strategies during daily tasks. To reinforce the concepts learned during the sessions, participants were also provided with supplementary educational brochures. While continuous on-site observation of the participants’ workplace practices was not feasible, the translation of these ergonomic principles into daily habits was evaluated by comparing pre-intervention (baseline) and post-intervention assessment scores. This method allowed for the objective confirmation of changes in ergonomic practices without disrupting the participants’ regular work routines.

The supervised exercise programme was performed three times per week for 12 weeks, providing 36 planned sessions. Each 45–60 min session consisted of approximately 10 min of warm-up, 30 min of postural control and strengthening exercises, 10 min of stretching, and 5 min of relaxation or cool-down. Postural control exercises were performed for 3 sets of 10–15 repetitions, with 60 s of rest between sets. Stretching exercises were held for 30 s and repeated 3 times for each muscle group. Exercise intensity was regulated using the Borg Rating of Perceived Exertion scale (6–20), with a target intensity of 12–14. The programme was progressed every 2 weeks by increasing resistance and exercise complexity, according to participant tolerance and symptom response. Exercises were modified or temporarily discontinued if pain exceeded 3/10 or if adverse symptoms occurred. Attendance and completion were recorded using an exercise log.

Attendance and intervention delivery were documented using an exercise log. The treating physiotherapist recorded session completion, exercise modifications, missed sessions, reasons for non-attendance, and protocol deviations. Participants in both groups were instructed not to initiate any new structured exercise programme, physiotherapy, or other treatment for their musculoskeletal complaints during the 12-week study period. The ergonomics-only group did not participate in the supervised exercise sessions. Any additional treatment, independent exercise, pain exacerbation, adverse event, or reason for intervention discontinuation was recorded throughout follow-up.

### 2.6. Outcome Measures

Validated Turkish-language versions of all multi-item questionnaires were used. Pain intensity, the primary outcome, was assessed using the Visual Analogue Scale (VAS), a widely used and psychometrically supported 0–10 measure on which higher scores indicate greater pain [18]. Musculoskeletal symptoms were assessed using the Turkish version of the Extended Nordic Musculoskeletal Questionnaire (NMQ-E), which has demonstrated adequate internal consistency, construct validity, and test–retest reliability; the total symptom score represented the number of affected body regions from 0 to 9 [19]; the total symptom score was the number of regions affected (0–9). Fatigue was assessed with the Fatigue Severity Scale (FSS) [20], sleep quality with the Pittsburgh Sleep Quality Index (PSQI) [21], and occupational burnout with the Maslach Burnout Inventory (MBI), comprising emotional exhaustion, depersonalization, and personal accomplishment subscales [22]. All measures were obtained at baseline and after the 12-week programme.

### 2.7. Statistical Analysis

All analyses were performed in IBM SPSS Statistics version 26 (IBM Corp., Armonk, NY, USA). Normality of within-pair differences and group distributions was assessed with the Shapiro–Wilk test [23]. Continuous variables are summarized as the mean (standard deviation) and median [interquartile range]; categorical variables are summarized as n (%). The pre-specified primary contrast was the between-group difference at 12 weeks, estimated by analysis of covariance (ANCOVA), with the post-intervention score as the outcome, group as the fixed factor, and the corresponding baseline value as the covariate. Effects are reported as baseline-adjusted mean differences with 95% confidence intervals and two-sided *p*-values. The adjusted mean difference and its confidence interval were considered the principal measures of effect magnitude and precision. Baseline adjustment was pre-specified because the groups differed at baseline on the NMQ-E total score. Within-group change was a secondary contrast (paired *t*-test or Wilcoxon signed-rank test, normality-guided), and between-group change was corroborated by the Mann–Whitney U test on change scores. Regional Nordic symptoms (binary) were compared within groups using the McNemar test. A clinically anchored responder analysis compared the proportions achieving ≥30% and ≥50% reductions in VAS between groups (Fisher’s exact test), with response thresholds based on established recommendations [24,25]. Pain intensity (VAS) was the primary outcome and all other measures were secondary; given the exploratory nature of the secondary endpoints, *p*-values are presented without multiplicity correction and interpreted cautiously. Analyses followed the intention-to-treat principle; no participant was lost after randomization. Two-sided *p* < 0.05 was considered statistically significant.

## 3. Results

In total, 52 male construction workers were randomized and all of them completed the 12-week protocol (Figure 1). Intervention attendance and fidelity were evaluated using the exercise logs and physiotherapist records. Participants assigned to the ergonomics-plus-exercise group demonstrated high adherence to the supervised programme, and the intervention was delivered according to the planned frequency and session duration. Missed sessions were limited and were primarily related to work schedules or personal reasons; no participant discontinued the programme because of exercise-related symptoms. No major protocol deviations or meaningful contamination between groups were identified. Participants did not report initiating additional physiotherapy or a new structured exercise programme during follow-up. No serious adverse events, intervention-related work injuries, or exercise-related pain exacerbations requiring treatment modification or discontinuation were recorded. The groups were comparable at baseline in age, body-mass index, occupation, and education (all *p* > 0.05; Table 1). The baseline demographic and clinical characteristics are presented descriptively in Table 1. The ergonomics-plus-exercise group had a higher median NMQ-E total score than the ergonomics-only group at baseline (five vs. four symptomatic regions). This difference was addressed by including the baseline NMQ-E score as a covariate in the corresponding ANCOVA model.

The primary baseline-adjusted analysis did not demonstrate a statistically significant difference in post-intervention VAS between the ergonomics-plus-exercise and ergonomics-only groups (adjusted mean difference −0.34, 95% CI −0.99 to 0.32, *p* = 0.303). Similarly, no statistically significant between-group differences were identified for fatigue, sleep quality, NMQ-E total score, emotional exhaustion, or depersonalization. Within-group pre–post changes are reported as secondary descriptive analyses and should not be interpreted as evidence of the efficacy of either intervention in the absence of an untreated control group.

Both arms improved significantly from baseline to 12 weeks. Pain (VAS) decreased from a median of five to two in the ergonomics-plus-exercise group and from six to two in the ergonomics-only group (both *p* < 0.001), with large within-group effects. Parallel within-group reductions were observed for fatigue (FSS), sleep quality (PSQI total), and musculoskeletal symptom burden (NMQ-E total) in both arms (all *p* < 0.001; Table 2). Personal-accomplishment scores increased significantly in both arms (both *p* < 0.001), whereas emotional exhaustion and depersonalization did not change in either group (all *p* > 0.10).

Crucially, baseline-adjusted between-group comparisons showed no incremental benefit of added exercise over ergonomics training alone. ANCOVA revealed no significant between-group difference for pain, fatigue, sleep, NMQ-E total score, emotional exhaustion, or depersonalization. A baseline-adjusted difference in personal accomplishment favoured the ergonomics-only group (adjusted mean difference −0.74, 95% CI −1.34 to −0.13, *p* = 0.018). This secondary finding was not reproduced in the change-score analysis (*p* = 0.465) and should be interpreted cautiously because no pre-specified clinically important threshold was available and multiple secondary outcomes were evaluated. The null between-group findings were corroborated by the change-score comparisons (all *p* > 0.05).

Exploratory responder analyses did not identify a statistically significant between-group difference at either the 30% or 50% pain-reduction threshold. In a responder analysis, a higher proportion of this arm achieved at least a 30% reduction in pain (96% vs. 77%; odds ratio 7.5), a non-significant trend (Fisher exact *p* = 0.099); the difference disappeared at the ≥50% threshold (73% vs. 58%, *p* = 0.382).

Regional musculoskeletal symptom prevalence declined in both arms (Table 3). In the ergonomics-plus-exercise group, significant within-group reductions occurred at the neck, shoulder, elbow, hand/wrist, low back, knee, and ankle/foot (all *p* ≤ 0.031). In the ergonomics-only group, significant reductions occurred at the shoulder, elbow, and hand/wrist (all *p* ≤ 0.039), with non-significant trends at the remaining regions. It should be noted that the regional analyses across the nine anatomical sites were conducted as secondary evaluations without multiplicity correction. Consequently, the statistically significant differences (*p* < 0.05) observed in specific regions are presented as exploratory and preliminary patterns rather than definitive anatomical outcomes.

## 4. Discussion

The principal finding of this randomized active-comparator trial was that adding supervised exercise to ergonomics education did not produce a statistically significant baseline-adjusted improvement in pain compared with ergonomics education alone. No statistically significant incremental benefit was identified for fatigue, sleep quality, overall musculoskeletal symptom burden, emotional exhaustion, or depersonalization. These findings indicate that superiority of the combined programme was not demonstrated; however, given the limited sample size and imprecise estimates, they should not be interpreted as evidence that the interventions were equivalent or that exercise has no potential benefit. Although both groups showed pre–post improvements, these within-group changes cannot establish intervention efficacy because the study did not include an untreated or usual-care group. Natural symptom fluctuation, regression to the mean, repeated measurement, treatment expectations, therapist contact, and research-participation effects may have contributed. The responder and regional analyses were secondary and exploratory and should similarly not be regarded as confirmatory evidence of an exercise-specific effect.

The isolated baseline-adjusted difference in personal accomplishment favouring the ergonomics-only arm should not be dismissed solely because it was contrary to the hypothesized direction. All participants worked at least 45 h per week, and the addition of three 45–60 min exercise sessions per week may have been perceived as an extra time, physical, or recovery demand. It is therefore possible that workers receiving ergonomics training alone experienced slightly less intervention burden and consequently reported a greater sense of occupational accomplishment. Nevertheless, this interpretation remains speculative because intervention-related burden, time pressure, and session-specific fatigue were not directly evaluated, and the finding was not supported by the change-score analysis.

Because both groups received an active intervention (ergonomics training) and the trial included neither an untreated control nor participant or therapist blinding, the parallel within-group improvements cannot be attributed solely to the interventions Natural symptom fluctuation and regression to the mean may have contributed to the observed changes. Research-participation effects may also have influenced participant-reported outcomes [26]. Moreover, because the primary outcome (VAS) and all secondary outcomes were participant-reported, knowledge of treatment allocation may have influenced ratings through treatment expectations, reporting or social-desirability bias, and differential contact with the treating physiotherapist. The additional supervised contact received by the exercise group may also have introduced an attention effect. Although coding and analyzing the dataset without group labels reduced analytic bias, it could not eliminate these performance and reporting effects. The improvements are consistent with meta-analytic evidence that broadly defined ergonomic interventions can reduce work-related musculoskeletal pain, although effects vary across anatomical regions and evidence regarding long-term effectiveness remains limited [27].

The absence of a measurable incremental effect of added exercise contrasts with parts of the literature. Network meta-analytic evidence suggests that exercise, alone or combined with education, can reduce episodes of low back pain and related absenteeism [28], and structured workplace exercise programmes have improved pain and functional capacity in office workers [10]. Reviews of combined ergonomic and physical-activity interventions, however, report considerable heterogeneity and variable effect sizes [29], and behaviour-focused interventions such as manual-handling training have not consistently prevented back pain when rigorously evaluated [30]. Several features of the present trial may explain the null incremental effect: the relatively short 12-week horizon; a possible ceiling effect, since both arms already received an active ergonomics intervention; a potentially insufficient or insufficiently progressive training stimulus, particularly because the programme emphasized postural control, stretching, and relaxation exercises without quantified intensity or progressive resistance loading; and a sample size powered to detect within-group rather than between-group differences. Implementation processes are considered particularly important in construction ergonomics, and construction-based trials have incorporated process evaluations to identify potential barriers and facilitators to intervention uptake [31].

An optimal exercise prescription specifically for construction workers has not yet been established. Construction-based studies have used protocols ranging from 10 min warm-up sessions performed on each working day to individually tailored aerobic and strengthening exercise performed for approximately 20 min three times per week over 12 weeks [32,33]. More general resistance-training guidance supports training the major muscle groups at least twice weekly and emphasizes individualized, progressive loading rather than a single fixed exercise volume [34]. Although not conducted specifically in construction workers, a recent randomized controlled trial showed that a targeted exercise programme reduced work-related neck and upper-back pain and improved mood across various occupational populations [35]. For workers already exposed to at least 45 h per week of physically demanding occupational activity, exercise frequency, intensity, and duration should therefore be adjusted according to baseline capacity, workday fatigue, and recovery. Two to three relatively brief and task-specific sessions per week, delivered at a moderate and progressively adjusted intensity with adequate recovery, may be more feasible than simply increasing total exercise duration. In the present study, total scheduled session time was not low; however, the absence of quantified intensity and progressive resistance loading may have limited the additional physiological stimulus.

Exploratory responder analysis showed a numerically higher proportion of participants achieving at least a 30% reduction in pain in the ergonomics-plus-exercise group than in the ergonomics-only group (96% vs. 77%; odds ratio, 7.5). However, this between-group difference was not statistically significant (p = 0.099), and the difference was smaller at the ≥50% threshold (73% vs. 58%, p = 0.382). The ≥30% and ≥50% thresholds reflect clinically important individual-level changes in pain intensity [24,25] and should not be interpreted as establishing the clinical importance of the between-group treatment effect. Therefore, these findings should be considered exploratory and hypothesis-generating. Although the numerically greater improvements in pain, fatigue, and regional symptom burden observed in the exercise group may justify further investigation, they do not provide confirmatory evidence of an exercise-specific benefit.

Several interacting factors may explain why the addition of exercise did not produce a statistically significant incremental effect. First, ergonomics training was itself an active intervention and resulted in substantial improvement, potentially leaving limited scope for an additional between-group effect. Second, participants were exposed to at least 45 h per week of physically demanding work, and the additional exercise sessions may have competed with recovery time or may have been perceived as an added burden. Third, the programme focused primarily on postural control, stretching, and relaxation, and the physiological stimulus may have been insufficient if exercise intensity and progression were not systematically increased. Fourth, the sample may have been insufficiently powered to detect small incremental differences between two active interventions. Finally, participant expectations, therapist contact, adherence, and broader research-participation effects may have contributed to improvement in both groups. These mechanisms were not measured directly and should therefore be interpreted as plausible explanations rather than confirmed causes.

Taken together, these findings position ergonomics training as a feasible backbone intervention in the construction setting, while indicating that the additional, clinically meaningful benefit of a short exercise programme remains uncertain. Future trials should be adequately powered for between-group comparisons, incorporate an untreated or attention-control arm and blinded outcome assessment, monitor exercise adherence objectively, and extend follow-up to evaluate the durability of effects.

### Limitations

This study has several limitations. The interventions were conducted in a single construction company and included only male workers, limiting generalizability. The active-comparator design did not include usual-care/no-intervention or exercise-only groups; therefore, the absolute and independent effects of ergonomics education and exercise could not be determined, and the within-group improvements cannot be causally attributed to either intervention.

Participants and the treating physiotherapist were not blinded, and the same physiotherapist delivered the interventions and administered the outcome questionnaires. Moreover, all outcomes were participant-reported, increasing the possibility of expectation, recall, reporting, and social-desirability bias. Although group labels were masked during analysis, this procedure could not control bias arising during intervention delivery or outcome collection. Future studies should include blinded assessors and objective functional and occupational outcomes.

The work-relatedness of the participants’ symptoms was not formally established through clinical diagnosis, occupational exposure assessment, or confirmation of a temporal relationship with work. In addition, the ergonomics component consisted of education only and did not include workplace modification, task redesign, or objective verification of behavioural or biomechanical changes. The sample-size calculation assumed a large effect and was not specifically based on the baseline-adjusted between-group difference in VAS; thus, the study may have been underpowered to detect small or moderate incremental effects, and the null findings should not be interpreted as evidence of equivalence. The 12-week follow-up also limits conclusions regarding long-term effectiveness. Finally, the modest baseline imbalance in NMQ-E was statistically adjusted, adherence and fidelity were monitored through logs rather than independent methods, and the responder and regional analyses should be considered exploratory because multiple secondary comparisons were performed.

A notable limitation of our study concerns the statistical approach to the secondary regional analyses. We conducted multiple hypothesis tests for nine separate anatomical regions within each intervention arm without applying a multiplicity correction. Under these circumstances, there is an increased risk of Type I error, meaning some of the observed *p*-values below 0.05 may have arisen by chance in the absence of a true regional effect. Because these analyses are highly multiple and based on small cell counts, the reported regional significance should be interpreted solely as exploratory rather than confirmatory.

## 5. Conclusions

Among construction workers with persistent musculoskeletal complaints, this trial did not demonstrate a statistically significant incremental benefit of adding a 12-week supervised exercise programme to ergonomics education. The findings should not be interpreted as evidence of equivalence or absence of a potentially meaningful exercise effect because the study was limited by its sample size, active-comparator design, unblinded participant-reported outcomes, and incomplete assessment of intervention exposure and potential contamination. Larger trials with clearly documented allocation concealment, objective outcomes, adherence and safety monitoring, and usual-care and exercise-only comparator groups are required.

## Figures and Tables

**Table 1:** Baseline characteristics of the participants.

Characteristic	Ergonomics + Exercise (n = 26)	Ergonomics Only (n = 26)
Age, years, mean	38.5 ± 8.28	40.08 ± 9.8
Body-mass index, kg/m^2^	25.71 ± 1.35	25.16 ± 1.72
**Occupation, n (%)**		
Painter	4 (15.4)	4 (15.4)
Ironworker	4 (15.4)	3 (11.5)
Electrician	4 (15.4)	7 (26.9)
Formworker	6 (23.1)	5 (19.2)
Plasterer	6 (23.1)	3 (11.5)
Plumber	2 (7.7)	4 (15.4)
**Education, n (%)**		
Primary	6 (23.1)	5 (19.2)
Secondary	6 (23.1)	4 (15.4)
High school	7 (26.9)	10 (38.5)
University	7 (26.9)	7 (26.9)

Between-group comparisons by independent *t*-test (age), and chi-square/Fisher’s exact test (categorical).

**Table 2:** Within- and between-group results for continuous outcomes.

Outcome	Group	Pre	Post	Within *p*	Adjusted Between-Group Difference [95% CI]	Between *p*
VAS (0–10)	Ergo + Exercise	5 [4–7]	2 [1–3]	<0.001	−0.34 [−0.99, 0.32]	0.303
	Ergo only	6 [4–8]	2 [1–5]	<0.001		
FSS (1–7)	Ergo + Exercise	3.77 [2.1–4.4]	2.0 [1.2–2.5]	<0.001	−0.24 [−0.57, 0.10]	0.163
	Ergo only	3.33 [2.6–4.7]	2.38 [1.2–3.3]	<0.001		
PSQI total (0–21)	Ergo + Exercise	6 [2.3–11]	2 [0.3–6.3]	<0.001	−0.03 [−0.77, 0.72]	0.946
	Ergo only	6 [2–14]	2.5 [1–5.8]	<0.001		
NMQ-E total (0–9) †	Ergo + Exercise	5 [4–6]	3 [2.3–4]	<0.001	+0.29 [−0.42, 1.00]	0.422
	Ergo only	4 [3.3–5]	2 [2–4]	<0.001		
MBI emotional exhaustion	Ergo + Exercise	10.5 [7.5–13.8]	12.0 [7.3–16.8]	0.169	+1.04 [−1.37, 3.45]	0.389
	Ergo only	10.5 [7.3–13.0]	10.0 [2.3–18.8]	0.989		
MBI depersonalization	Ergo + Exercise	4.5 [3.3–5.8]	2.5 [1.0–6.8]	0.107	−0.56 [−1.69, 0.56]	0.317
	Ergo only	5.0 [3.3–6.8]	5.5 [1.3–8.0]	0.806		
MBI personal accomplishment	Ergo + Exercise	25.5 [23.0–29.0]	30.0 [27.3–32.0]	<0.001	−0.74 [−1.34, −0.13]	0.018
	Ergo only	26.5 [24.0–29.0]	30.5 [29.0–32.0]	<0.001		

Values are presented as median [interquartile range]. Within-group *p*-values were obtained using paired *t*-tests or Wilcoxon signed-rank tests, as appropriate based on distributional assumptions. Between-group differences represent baseline-adjusted ANCOVA estimates for the ergonomics-plus-exercise group minus the ergonomics-only group, with 95% confidence intervals. † The baseline imbalance in the NMQ-E total score was addressed using baseline-adjusted ANCOVA. VAS—Visual Analogue Scale; FSS—Fatigue Severity Scale; PSQI—Pittsburgh Sleep Quality Index; NMQ-E—Extended Nordic Musculoskeletal Questionnaire; MBI—Maslach Burnout Inventory.

**Table 3:** Regional musculoskeletal symptom prevalence and within-group change.

Region	Ergo + Exercise Pre → Post (%)	*p*	Ergo Only Pre → Post (%)	*p*
Neck	69 → 39	0.008	50 → 31	0.125
Shoulder	65 → 35	0.008	54 → 27	0.016
Upper back	54 → 46	0.500	46 → 23	0.070
Elbow	46 → 15	0.008	54 → 27	0.039
Hand/wrist	58 → 23	0.004	50 → 12	0.002
Low back	81 → 58	0.031	62 → 42	0.125
Hip/thigh	54 → 35	0.062	50 → 31	0.180
Knee	62 → 39	0.031	54 → 35	0.125
Ankle/foot	54 → 23	0.021	42 → 31	0.375

*p*-values refer to within-group (pre–post) comparisons using the exact McNemar test.

## Data Availability

The datasets generated and/or analyzed during the current study are not publicly available due to patient privacy, confidentiality, and institutional ethical restrictions. De-identified data may be made available from the corresponding author upon reasonable request, subject to approval by the relevant institutional ethics committee and applicable data-sharing regulations.
